# Thermal and cardiovascular heat adaptations in active adolescents following summer

**DOI:** 10.1080/23328940.2024.2347161

**Published:** 2024-05-05

**Authors:** Harry A. Brown, Thomas H. Topham, Brad Clark, Andrew P. Woodward, Leonidas G. Ioannou, Andreas D. Flouris, Richard D. Telford, James W. Smallcombe, Ollie Jay, Julien D. Périard

**Affiliations:** a Research Institute for Sport and Exercise Science (UCRISE), University of Canberra, Bruce, ACT, Australia; b Faculty of Health, University of Canberra, Bruce, ACT, Australia; c FAME Laboratory, Department of Physical Education and Sport Science, University of Thessaly, Trikala, Greece; d Heat and Health Research Incubator, Faculty of Medicine and Health, The University of Sydney, Sydney, NSW, Australia

**Keywords:** Heat adaptation, pediatric, seasonal, sweating, thermoregulation

## Abstract

This study aimed to investigate seasonal heat acclimatization in active adolescents following summer. Fifteen (5 females) active adolescents (14.6 ± 1.0 y) completed a 45-min heat response test (HRT) walking at 60% $\dot{V}$O_2peak_ in 40°C and 30% relative humidity before and after summer (i.e. November 2022 and March 2023). During the HRT, gastro-intestinal temperature (T_gi_), skin temperature (T_sk_), heart rate, local sweat rate (LSR) and whole-body sweat loss (WBSL) were recorded. Carbon monoxide rebreathing and dual-energy X-ray absorptiometry scans determined resting hematological measures and body composition. Participants completed physical activity (PA) diaries and wore an accelerometer for two one-week periods (pre- and post-summer). Daytime wet-bulb globe temperature (WBGT) was calculated for each summer day. Data are presented as posterior mean and 90% credible intervals. Participants reported 7 ± 4 h·wk^−1^ of outdoor PA, and daytime WBGT was 21.2 ± 4.6°C. Following summer, resting T_gi_ and heart rate were reduced by 0.2°C [−0.3, −0.1; probability of direction = 99%] and 7 beats·min^−1^ [−10, −3; 100%], respectively. During the HRT, there was an earlier onset of sweating (−0.2°C [−0.3, −0.0; 98%]), an attenuated rise of T_gi_ (0.2°C [−0.5, 0.0; 92%]) and mean T_sk_ changed by −0.2°C [−0.5, 0.1; 86%]. There was minimal evidence for heat adaptations in LSR or WBSL, hematological parameters or perceptual measures. This is the first study to demonstrate seasonal heat adaptations in active adolescents. Reductions in resting T_gi_ and exercising T_sk_ and a lower T_gi_ at the onset of sweating were associated with a smaller rise in T_gi_ during the HRT following summer.

## Introduction

Physiological adaptations to environmental heat stress reduce thermal strain, support cardiovascular stability, and improve aerobic performance in the heat [1,2]. More specifically, a reduction in exercising core temperature and heart rate, an enhanced whole-body sweat rate (WBSR), and an earlier onset for sweating and skin vasodilation are induced following repeated exposures to a hot environment [3]. The induction of physiological heat adaptations is referred to as heat acclimation or acclimatization, with the former associated with repeated exposures to an artificial environment and the latter involving exposures to the natural environment. Individuals who reside in regions that experience seasonal temperature changes with warm summers can potentially adapt to the heat, with the magnitude of adaptation dependent on the environmental characteristics, as well as the type, frequency and timing of outdoor exposures [4].

Seasonal heat acclimatization has been shown to attenuate the rise in core temperature (~0.2°C) and heart rate (~6 beats∙min^−1^) during laboratory-based heat response tests, as well as improve the sudomotor response (i.e. greater maximum WBSR) [5,6]. However, seasonal heat acclimatization has only been assessed in adult populations [5,7,8]. Dougherty et al. [9] monitored the induction of heat adaptations in 9- to 12-year-old boys during a six-day exercise-based heat acclimation protocol, which enhanced WBSR (~0.11 L·h^−1^) and reduced exercising core temperature (~0.2°C). Thus demonstrating that laboratory-based heat acclimation can be induced in a pediatric population [9]. In response to environmental heat stress, physical (i.e. body surface area to mass ratio) and physiological differences (i.e. sweating capacity) between children and adults can potentially lead to differences in thermoregulation [10,11]. However, when assessing these differences, the prescribed exercise intensity is vital to ensure an unbiased comparison [12]. Notwithstanding, following repeated bouts of heat exposures it has been suggested that children adapt more slowly and to a smaller magnitude [13], and experience less physiological changes across seasons [14], than adults. As such, the natural attainment of heat adaptations from the summer months within younger populations requires attention.

During summer, when schools are out of session and the days are longer with favorable weather conditions for outdoor activities, adolescents demonstrate an ~2.3 h∙wk^−1^ increase in outdoor physical activity, compared to winter [15]. Given that seasonal heat acclimatization is influenced by the duration and intensity of outdoor physical activity, as well as the timing of environmental exposures [4], the additional exposure to hot outdoor environmental conditions during exercise and physical activity may present adolescents with a greater opportunity to acclimatize to the summer heat. Developing our understanding of the magnitude of heat acclimatization induced in adolescents throughout summer has implications for those involved in youth sports, both from a performance and safety perspective [16].

Therefore, the aim of this study was to evaluate select markers (i.e. core temperature, heart rate, plasma volume) of heat adaptation in adolescents before and after summer, and to document the factors (i.e. environmental conditions, physical activity characteristics) that influence the magnitude of change.

## Methods

### Participants

Fifteen (5 girls) active adolescents between 12 and 16 years of age were recruited (Table 1). Before the commencement of any testing, study protocols were explained in full, and both participants and their guardians provided written assent and consent, respectively. The study was approved by the University of Canberra Research Ethics Committee (Project ID: 20214535), and all procedures conformed to the standard of the *Declaration of Helsinki*.

**Table 1.** Participant characteristics pre- and post-summer, including $\dot{V}$O_2peak_ and reported Tanner stage.

Characteristic	Pre-summer	Post-summer
Age (y)	14.6 ± 1.0	14.9 ± 1.0
Height (m)	1.70 ± 0.08	1.71 ± 0.08
Body mass (kg)	56.84 ± 9.05	58.22 ± 9.25
Body fat (%)	18.4 ± 4.9	18.2 ± 4.8
$\dot{V}$O_2peak_ (mL·kg^−1^·min^−1^)	60.4 [56.2, 64.6]	61.6 [57.4, 65.8]
Tanner stage (*n* = 15)		
Pre-pubertal (Stage 1)	0	0
Mid-pubertal (Stage 2–4)	12	11
Late-pubertal (Stage 5)	3	4

Data presented as mean ± standard deviation, except for $\dot{V}$O_2peak_ presented as posterior mean and 90% credible interval, and Tanner stage presented as counts. $\dot{V}$O_2peak_; peak oxygen consumption.

### Experimental design

Initial testing took place in November 2022, prior to the commencement of summer (i.e. December 1^st^) in Canberra, Australia. Participants were asked to return after summer in March 2023 to repeat all testing procedures. This region of Australia is temperate, does not have a dry season, but does have warm summers [17]. Each testing period included an incremental exercise test to ascertain peak oxygen consumption ($\dot{V}$O_2peak_), a dual-energy X-ray absorptiometry (DXA) scan to determine body composition, and a heat response test to establish physiological responses to exercise-heat stress. All participants completed the Tanner scale to self-report maturation stage [18]; female participants also completed a self-reported menstrual cycle questionnaire to ensure heat response tests took place during the same menstrual phase within participants. Before each visit to the laboratory, participants were asked to refrain from strenuous exercise and caffeine for at least 24 h.

### Incremental exercise test and carbon monoxide re-breathing

Participants completed five submaximal stages on a motorized treadmill (Pulsar 3p, H/P/Cosmos, Nussdorf-Traunstein, Germany), each lasting 4 min. The speed for the submaximal stages was kept constant at a comfortable individualized walking pace (5.0–6.5 km·h^−1^). The starting gradient was 2% and increased by 2% (percentage points) for each stage. After 20 min of submaximal walking, participants rested for 10 min and then ran at a pre-selected speed (10.0–15.0 km·h^−1^) to volitional exhaustion. During the run to exhaustion, the initial gradient of the treadmill was 0% and increased by 1% (percentage points) each min. The treadmill gradient that equated to 60% $\dot{V}$O_2peak_ while walking was calculated following the incremental run to volitional fatigue. Participants were equipped with a mouthpiece (Hans Rudolph Inc., Shawnee, KS, USA) to allow for the analysis of expired gases (TrueOne, Parvomedics, Sandy, UT, USA) during the submaximal walk and incremental run. Heart rate was recorded throughout, whilst the rating of perceived exertion (RPE) was recorded in the final 30 s of each submaximal stage and the end of the incremental exercise test using a pictorial OMNI scale (11 point scale where 0 = extremely easy and 10 = extremely hard [19]). Participants then rested for 30 min before completing a carbon monoxide (CO) re-breathing procedure [20]. On completion of testing, participants were provided with a self-report physical activity diary and a waist-worn accelerometer (Actigraph, wGT3X-BT, Pensacola, Florida, USA) to record physical activity patterns for one week pre- and post-summer.

The modified CO re-breathing procedure to calculate hemoglobin mass, plasma volume and blood volume was used and has been described in detail previously [20,21]. Briefly, participants rested for 30 min to allow the stabilization of plasma volume before fingertip capillary blood samples were taken to ascertain carboxyhemoglobin (HbCO) concentration (1 × 200 uL capillary tube) and hematocrit (4 × 75 uL capillary tubes). Participants then fully exhaled into a CO gas meter to record baseline CO in the lungs (CO-220 Carbon Monoxide Meter, Fluke, Australia). The 2 min CO re-breathing procedure began when participants exhaled to the residual volume before inhaling a mixture of 3-L Oxygen and 0.8 mL·kg^−1^ CO [21]. This mixture was then held in the lungs for 10 s before normal breathing commenced for 110 s. Following a full exhalation into a spirometer where CO not absorbed was analyzed, HbCO was measured again via a fingertip capillary blood sample (~7 min after the procedure began). Following the calculation of hemoglobin mass, intravascular volumes were estimated [22]. During pre-summer measures, CO re-breathing was completed in duplicate with a typical error in our laboratory of 1.7%.

### DXA scan

Following an overnight fast, a DXA scan (GE-LUNAR Prodigy module, GE Medical Systems, Madison, WI) was conducted to determine body composition. Participants arrived in loose fitting clothing and before each scan, jewelry and metal objects were removed. All scans were completed using the same scanner and by the same accredited densitometrist.

### Heat response test

Approximately 8 h before each heat response test participants swallowed an ingestible telemetric capsule (e-Celsius, BodyCap, France) to measure gastro-intestinal temperature (T_gi_) at a sampling rate of 15 s [23]. On arrival at the laboratory, participants provided a urine sample for measurement of urine specific gravity (USG) to ascertain hydration status with a handheld refractometer (PEN-Urine S.G., Atago Co. Ltd., Tokyo, Japan). If USG was > 1.025 [24], participants drank 5 mL·kg^−1^ of room temperature water during instrumentation, ~30 min before the commencement of exercise. Participant clothing was weighed before a clothed body mass was recorded (KW-4050-150+, KW Industrial Platform Scale, VIC, Australia). Participants were then instrumented with four skin temperature sensors (iButtons, Maximum Integrated Products, San Jose, CA, USA) to calculate weighted mean skin temperature (T_sk_: chest (30%), shoulder (30%), thigh (20%) and calf (20%) [25]), two absorbent patches (6 × 7 cm; Tegaderm; 3 M, USA) to measure sweat sodium concentration ([Na^+^]) via a portable sweat test system (MX3 Diagnostics Inc., Melbourne, Australia), and a heart rate monitor chest strap (HRM Dual, Garmin International Inc, Olathe, Kansas, USA). The two absorbent patches for sweat [Na^+^] analysis were placed on the right side of the upper back and the forearm. Following instrumentation, baseline measures were recorded during 10 min rest in a temperate environment (22.3 ± 1.1°C air temperature, 44.9 ± 6.6% relative humidity (RH)).

Participants then entered an environmental chamber (QRA International Pte Ltd, Singapore) set to hot conditions (40.0 ± 0.7°C, 30.5 ± 1.4% RH) and sat on a chair for further instrumentation. Local sweat rate (LSR) was measured using two ventilated capsules (4.0 cm^2^) affixed to the skin using adhesive discs (3 M Health Care, Neuss, Germany) and surgical tape (Transpore, 3 M, London, ON, Canada). One capsule was placed on the left side of the upper back ~5 cm above the scapular spine, and the other was placed ~5 cm distal to the antecubital fossa. Anhydrous air was passed through the capsule at a rate of 500 mL·min^−1^ with the humidity of effluent air measured at 0.2 Hz via a capacitance hygrometer (HMT333, Vaisala, Vantaa, Finland). LSR was calculated as the product of vapor concentration and flow rate.

Following instrumentation in the environmental chamber, participants rested for 10 min before walking for 45 min at a speed (6.1 ± 0.2 km∙h^−1^) and gradient (10.2 ± 2.2%) equivalent to 60% $\dot{V}$O_2peak_. During the first 15 min of the walk, expired gas was analyzed for oxygen and carbon dioxide concentration to verify exercise intensity. T_gi_ and T_sk_ were recorded continuously, whilst heart rate, thermal perception (thermal comfort (7 point scale where 1 = too cool and 7 = much too warm [26]) and thermal sensation (7 point scale where 1 = cold and 7 = hot [27]) and RPE were recorded every 5 min. Following the 45 min of exercise, clothed body mass was recorded with clothing mass measured separately. Partitional calorimetry was used to assess human heat balance (See Supplementary File). Changes in body mass pre- and post-exercise were used to calculate whole-body sweat loss (WBSL), accounting for sweat trapped in clothing, and respiratory water loss [28]. LSR was plotted against time and analyzed using segmented regression to allow for the determination of each participant’s onset threshold for sweating (time in min referenced against exact T_gi_) [29] and sweating sensitivity (in mg·cm^−2^·min^−1^·min^−1^) [30].

### Environmental and physical activity characteristics

Mean daytime (08:00–18:00) wet-bulb globe temperature (WBGT) was calculated for the entirety of summer (i.e. December 2022 to February 2023) in the Australian Capital Territory, Australia, using meteorological data [31,32] as described previously [4,33]. Ambient temperature, RH, wind speed and cloud coverage data were obtained from the National Oceanic and Atmospheric Administration (http://www.ncei.noaa.gov/data/global-hourly), whilst solar radiation was computed [34,35].

Participants completed the physical activity diary for one week pre- and post-summer and were asked to provide general information regarding their level of activity (i.e. time of day, duration, location, and intensity). The intensity of physical activity was reported using a 0–10 RPE scale [36] and categorized (light, moderate, high) according to Exercise and Sport Science Australia guidelines [37]. Participants also wore an accelerometer for the week and were instructed to take it off for periods where the device could be damaged (i.e. bathing and contact sports). Accelerometers were initialized to 60 Hz samples and processed as 60 s epochs, with physical activity determined via the Freedson VM3 Combination equations using all three axes of movement (X, Y, Z) [38].

### Statistical analysis

T_gi_, T_sk_, heart rate and LSR at the back and forearm during the heat response tests were defined as response variables and analyzed using Bayesian hierarchical generalized additive models (HGAM) [39], with time, stage of summer (pre, post) and $\dot{V}$O_2peak_ as predictor variables. This analysis implements the effects of numeric predictors as non-parametric terms, with smooth forms determined from the data [40], and was selected due to the known non-linearity of the data during heat responses tests. A thin-plate regression spline allowed for two continuous variables to be captured [41]. RPE, thermal sensation, and thermal comfort were defined as integer response variables. The perceptual variables were then analyzed using Bayesian HGAM akin to the one implemented for continuous variables, but a beta-binomial family distribution was utilized. For resting T_gi_, resting T_sk_, resting heart rate, pre-trial USG, WBSL, sweat [Na^+^] at the back and forearm, the three hematological measures (blood volume, plasma volume and hemoglobin mass) and measures from the incremental exercise test (maximum heart rate, respiratory exchange ratio), Bayesian linear hierarchical models were implemented. The predictor variables were the stage of summer (pre, post) and $\dot{V}$O_2peak_. Each model included a participant-level intercept to account for repeated measures. Models were implemented with normally distributed priors intended to be weakly informative placed on the intercept. Flat priors were selected for the smooth terms [41], whilst a scaled prior was used on continuous variables [42].

Models were implemented using “brms” package [43] in R statistical software (v 4.2.1) [44]. Model convergence was assessed using the Rhat statistic, effective sample sizes and visual inspection of MCMC chains [45]. Data are presented as posterior means and 90% credible intervals [90% CrI], which represents a 90% probability that the estimate is within the interval, given the observed data, the model and the priors. The 90% CrI width was chosen as it provides greater computational stability than 95% CrI due to the latter relying only on 2.5% of the posterior draws [46]. Model estimates for each outcome measure were predicted using arithmetic means for each predictor variable. In addition to posterior means and 90% CrI, the probability of direction (Pd) was calculated using “BayestestR” [47], which summarizes the approximate posterior probability (%) of the estimate either being positive (Pd+) or negative (Pd-). Posterior means and CrI were visualized using “ggdist” [48] via “ggplot2” [49].

## Results

### Incremental exercise test

$\dot{V}$O_2peak_appeared similar between pre- and post-summer (1.2 mL·kg^−1^·min^−1^[−0.5, 2.8; 88%]; Table 1). The peak respiratory exchange ratio was 1.04 [1.03, 1.06] pre-summer and 1.04 [1.03, 1.06] post-summer, adifference of 0.00 [−0.02, 0.02; 54%]. Maximum heart rate was 200 beats·min^−1^[197, 204] pre-summer and 196 beats·min^−1^[193, 199] post-summer, a change of −4 beats·min^−1^[−6, −2; 100%].

### Resting measures

Resting T_gi_ and heart rate changed by −0.2°C [−0.3, −0.1; 99%] and −7 beats·min^−1^ [−10, −3; 100%], respectively (Table 2). Following summer there were no changes to resting T_sk_, or the three hematological measures (i.e. hemoglobin mass, plasma volume and blood volume; Table 2).

**Table 2.** Gastro-intestinal and skin temperature, heart rate, hemoglobin mass, and plasma and blood volume at rest pre- and post-summer.

Outcome variable	Pre-summer	Post-summer	Mean difference [90% CrI; Pd]
Gastro-intestinal temperature (°C)	37.28 [37.17, 37.39]	37.09 [36.98, 37.19]	−0.19 [−0.31, −0.07; 99%]
Heart rate (beats·min^−1^)	80 [75, 85]	73 [68, 78]	−7 [−10, −3; 100%]
Skin temperature (°C)	32.15 [31.78, 32.52]	32.14 [31.77, 32.50]	−0.02 [−0.45, 0.42; 52%]
Hemoglobin mass (g·kg^−1^)	11.6 [10.9, 12.3]	11.9 [11.2, 12.6]	0.3 [−0.6, 1.2; 73%]
Plasma volume (mL·kg^−1^)	47.5 [45.1, 49.9]	46.9 [44.5, 49.4]	−0.5 [−3.6, 2.5; 61%]
Blood volume (mL·kg^−1^)	78.8 [74.9, 82.7]	77.6 [73.7, 81.7]	−1.1 [−6.0, 3.9; 63%]

Data presented as posterior means and 90% credible intervals [CrI]. Model estimates were based on the arithmetic mean of $\dot{V}$O2peak. Pd; probability of direction.

### Heat response test

During the pre-summer heat response test mean $\dot{V}$O_2_ during the first 15 min of exercise was 36.2 mL·kg^−1^·min^−1^ [34.0, 38.4] and was 1.1 mL·kg^−1^·min^−1^ [−1.6, −0.5; 99%] lower post-summer. Pre-trial hydration status was 1.014 [1.010, 1.019] pre-summer and 1.018 [1.013, 1.023] post-summer. All heat response tests were determined to be uncompensable as the ratio between the rate of evaporation required for heat balance and the maximum rate of evaporation ranged between 1.4 and 2.2. During pre-summer testing one participant was removed from the environmental chamber after 33 min of exercise as their T_gi_ reached 39.5°C. Following summer, this participant completed the entirety of the heat response test whilst maintaining a T_gi_ <39.5°C. Their data from both pre- and post-summer remained in the analysis.

**Figure 1.**
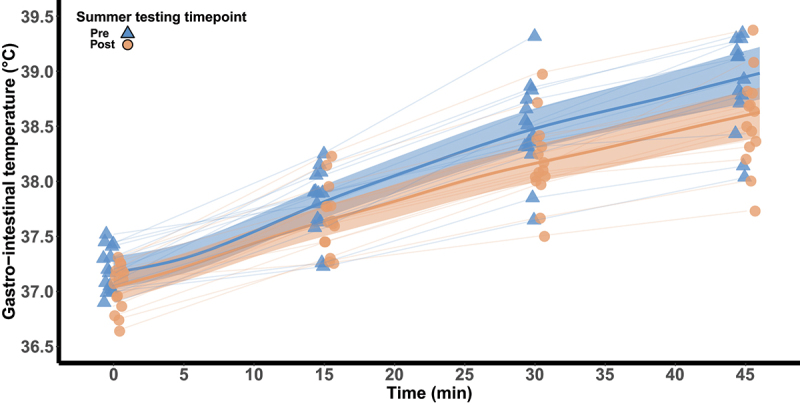
Gastro-intestinal temperature during 45 min of walking at 60% $\dot{V}$O_2peak_ in 40°C and 30% relative humidity pre- and post-summer. The thick lines represent the population predicted means surrounded by 90% credible intervals. Model estimates are based on the arithmetic mean of $\dot{V}$O_2peak_. The model was implemented using data from 5-min intervals. Observed data are visualized at 15-min intervals.

**Figure 2.**
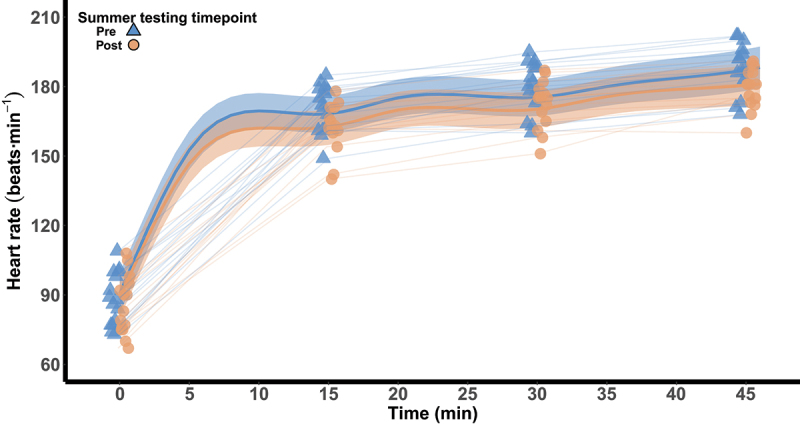
Heart rate during 45 min of walking at 60% $\dot{V}$O_2peak_ in 40°C and 30% relative humidity pre- and post-summer. The thick lines represent the population predicted means surrounded by 90% credible intervals. Model estimates are based on the arithmetic mean of $\dot{V}$O_2peak_. The model was implemented using data from 5-min intervals. Observed data are visualized at 15-min intervals.

T_gi_ from 0 to 45 min of exercise is visualized in Figure 1. The difference in the rise of T_gi_ and the difference in end-exercise T_gi_ between pre- and post-summer are shown in Table 3. T_sk_ during the heat response test was 36.3°C [36.1, 36.5] pre-summer and 36.1°C [35.8, 36.4] post-summer, a difference of −0.2°C [−0.5, 0.1; 86%]. Heart rate from 0 to 45 min of exercise is visualized in Figure 2. The difference in the rise of heart rate and the difference in end-exercise heart rate between pre- and post-summer are shown in Table 3. Mean heart rate during the heat response test pre-summer was 171 beats·min^−1^ [119, 193] and was 165 beats·min^−1^ [115, 185] post-summer, a difference of −6 [−14, 3; 87%].

**Table 3.** Gastro-intestinal temperature, heart rate, whole-body sweat loss, and sweat sodium concentration during the heat response test pre- and post-summer.

Outcome variable	Pre-summer	Post-summer	Mean difference [90% CrI; Pd]
Rise in T_gi_ (°C)	1.78 [1.57, 1.99]	1.56 [1.36, 1.76]	−0.22 [−0.48, 0.04; 92%]
End-exercise T_gi_ (°C)	38.95 [38.72, 39.18]	38.60 [38.37, 38.82]	−0.35 [−0.63, −0.07; 98%]
Rise in heart rate (beats·min^−1^)	97 [87, 106]	92 [83, 101]	−5 [−14, 4; 81%]
Heart rate (beats·min^−1^)	187 [178, 196]	180 [172, 189]	−7 [−16, 2; 89%]
WBSL (L)	0.78 [0.67, 0.90]	0.82 [0.70, 0.93]	0.03 [−0.06, 0.12; 73%]
Sweat [Na^+^] (mmol·L^−1^)			
Back	69	66	−3
	[60, 78]	[50] 75]	[−13, 6; 73%]
Forearm	65	57	−8
	[50] 72]	[51] 65]	[−14, −1; 97%]

Data presented as posterior means and 90% credible intervals [CrI]. Model estimates were based on the arithmetic mean of $\dot{V}$O_2peak_. T_gi_; Gastro-intestinal temperature, LSR; local sweat rate, Pd; probability of direction, Sweat [Na+]; sweat sodium concentration, WBSL; whole-body sweat loss.

T_gi_ at the onset of both back and forearm LSR was 37.2°C [37.1, 37.3] pre-summer and was 37.1°C [37.0, 37.1] post-summer, a difference of −0.2°C [−0.3, −0.0; 98%]. In contrast, sweating sensitivity appeared similar between pre- and post-summer (−0.01 mg·cm^−2^·min^−1^·min^−1^ [−0.02, −0.00; 95%]). Back LSR was 1.36 mg·cm^−2^·min^−1^ [1.16, 1.59] pre-summer, and changed by 0.06 mg·cm^−2^·min^−1^ [−0.15, 0.26; 68%] to 1.42 mg·cm^−2^·min^−1^ [1.23, 1.59] post-summer. Forearm LSR was 1.15 mg·cm^−2^·min^−1^ [1.00, 1.29] pre-summer, and changed by 0.04 mg·cm^−2^·min^−1^ [−0.08, 0.16; 72%] to 1.19 mg·cm^−2^·min^−1^ [1.05, 1.32] post-summer. Post-summer WBSL changed by 0.03 L [−0.06, 0.12; 73%], whereas sweat [Na^+^] at the back and forearm changed by −3 mmol·L^−1^ [−13, 6; 73%] and −8 mmol·L^−1^ [−14, −1; 97%], respectively (Table 3).

Five min into exercise RPE was 6.9 [6.1, 7.7] pre-summer and 5.1 [4.0, 6.3] post-summer, a difference of −1.8 [−2.9, −0.7; >99%]. Twenty-five min into exercise RPE was 6.9 [6.1, 7.7] and 5.9 [5.0, 6.8], a difference of −1.0 [−1.8, −0.2; 98%]. End-exercise RPE was 6.9 [6.1, 7.7] pre-summer and 6.6 [5.4, 7.6] post-summer, a difference of −0.4 [−1.4, 0.6; 72%]. Five min into exercise thermal sensation was 6.6 [6.3, 6.9] pre-summer and 6.5 [6.0, 6.8] post-summer, a difference of −0.1 [−0.5, 0.1; 77%]. Twenty-five min into exercise thermal sensation was 6.6 [6.3, 6.9] and 6.7 [6.3, 6.9], a difference of 0.0 [−1.6, 0.2; 58%]. End-exercise thermal sensation was 6.6 [6.3, 6.9] pre-summer and 6.7 [6.4, 6.9] post-summer, a difference of 0.1 [−0.1, 0.3; 80%]. Five min into exercise thermal comfort was 6.2 [5.8, 6.6] pre-summer and 6.0 [5.3, 6.4] post-summer, a difference of −0.3 [−0.7, 0.1; 84%]. Twenty-five min into exercise thermal comfort was 6.2 [5.8, 6.6] pre-summer and 6.2 [5.7, 6.5] post-summer, a difference of −0.0 [−0.3, 0.3; 54%]. End-exercise thermal sensation was 6.2 [5.8, 6.6] pre-summer and 6.3 [5.9, 6.7] post-summer, a difference of 0.1 [−0.2, 0.5; 76%].

### Environmental and physical activity characteristics

Pre-summer physical activity diaries showed that participants completed 12.4 ± 8.4 h·wk^−1^ of physical activity with 7.4 ± 3.6 h·wk^−1^ occurring outdoors. Of the outdoor physical activity, participants reported 2.2 ± 2.8 h·wk^−1^ to be at a low intensity, 2.7 ± 3.4 h·wk^−1^ as a moderate intensity, and 4.2 ± 2.1 h·wk^−1^ as a high intensity. Post-summer, participants reported 9.6 ± 6.3 h·wk^−1^, with 6.0 ± 3.5 h·wk^−1^ taking place outdoors. Of the outdoor physical activity, participants reported 1.6 ± 1.6 h·wk^−1^ to be at a low intensity, 3.0 ± 2.4 h·wk^−1^ as moderate intensity, and 3.8 ± 2.8 h·wk^−1^ as high intensity. Forty-nine percent of outdoor physical activity took place between 15:00 and 18:00, whilst the second most frequent (28%) period for performing outdoor physical activity was between 05:00 and 08:00. During the seven days of wearing the accelerometer the average moderate to vigorous physical activity pre- and post-summer were 2.3 ± 1.2 h·d^−1^ and 1.9 ± 1.1 h·d^−1^, respectively. All participants provided pre- and post-summer activity diaries, but accelerometer data was available for only 14 participants’ pre-summer.

**Figure 3.**
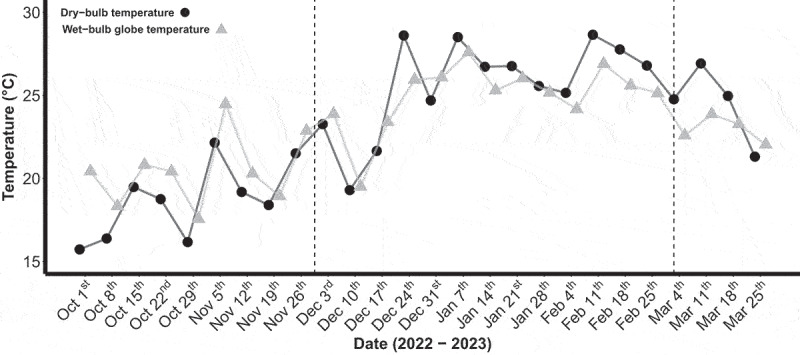
Mean maximum dry-bulb and wet-bulb globe temperature from October 1^st^ 2022 to March 31^st^ 2023. The individual data points represent weekly averages. The vertical dashed black lines represent the start and end of summer, respectively.

Mean daytime dry-bulb temperature, RH, black-globe temperature and WBGT during summer were 22.1 ± 5.3°C, 52.0 ± 19.9%, 33.8 ± 9.9°C and 21.2 ± 4.6°C, respectively. During the most common period for reported outdoor physical activity (i.e. 15:00–18:00), dry-bulb temperature, RH, black-globe temperature and WBGT were 24.3 ± 4.9°C, 42.8 ± 16.1%, 32.0 ± 8.0°C and 20.9 ± 3.9°C, respectively. In contrast, mean daily maximum dry-bulb temperature and WBGT during summer were 25.6 ± 4.9°C and 25.3 ± 4.6°C, respectively (Figure 3).

## Discussion

This study assessed the magnitude of seasonal heat acclimatization in active adolescents across the summer months in a temperate region that has warm summers. Heat adaptations were evident as reductions in resting T_gi_ of ~0.2°C and resting heart rate of ~7 beats·min^−1^ (Table 2). Similarly, the increase in T_gi_ during the heat response test and end-exercise heart rate were ~0.2°C and ~7 beats·min^−1^ lower following summer, respectively (Figures 1 and Figure 2). As such, our findings indicate that thermal and cardiovascular related heat adaptations were induced in adolescents during a summer characterized by a mean daytime WBGT of ~ 21.2°C. In contrast, changes in sudomotor (i.e. WBSL, LSR and sweat [Na^+^]), hematological (i.e. hemoglobin mass, plasma volume and blood volume; Table 2), and perceptual (i.e. RPE, thermal sensation and thermal comfort) measures were not evident.

Resting T_gi_ was ~0.2°C lower post-summer, which in comparison to a recent systematic review on seasonal heat acclimatization is larger than the average reduction (~0.1°C) [4], and more closely aligns with the magnitude of adaptation seen following exercise heat acclimatization [51]. Reductions in resting core temperature as large as ~0.2°C have been reported following summer in adult males in the Republic of Korea [7,52]. The authors attributed the reduction in resting core temperature in part to a reduced resting metabolic rate. Enhancements in blood volume have also been proposed to influence resting core temperature due to an increased cutaneous blood flow, which aids with heat flow from the core to the skin [53]; however, changes in blood volume were unclear in the current study. Interestingly, despite the active adolescents in the present study residing in an environment ~4°C WBGT less thermally stressful than the summers in the Republic of Korea, they experienced a comparable reduction in resting core temperature (~0.2°C) to that of the adult males (~0.1°C) [7,52]. Of note, both studies that took place in the Republic of Korea indicated that participants were exposed to outdoors conditions for >10 h·wk^−1^; however, the amount of time spent exercising and the timing of exposures is unclear. In a recent study of recreationally active adults in Canberra, Australia, we reported that exposure to a slightly warmer summer (~1°C WBGT) did not lead to a reduction in resting core temperature, which was likely due to the timing of outdoor exposures (i.e. most frequent time to train outdoors was 17:00–18:00) [54]. As such, the level of activity (~7 h∙wk^−1^) and the timing of outdoor exercise (49% between 15:00–18:00) undertaken by the adolescents, may have been sufficient to induce the reduction in resting T_gi_.

Reductions in the rise of T_gi_ (~0.2°C) and a lower end-exercise heart rate (~7 beats·min^−1^) during the heat response test indicate that the adolescents may have experienced a reduction in thermal strain and an enhanced cardiovascular stability following seasonal heat acclimatization (Figures 1 and Figure 2). Similar findings have been noted in endurance athletes in the North-Eastern United States completing ~6 h·wk^−1^ of outdoor training [55]. Of note, the active adolescents in the current study experienced a comparable reduction in mean exercising heart rate (~6 beats·min^−1^ compared to ~5 beats·min^−1^) and in the rise of T_gi_ (~0.2°C) to the endurance athletes, despite being exposed to a less thermally stressful environment (~2.5°C WBGT). Although it has been suggested that reductions in exercising heart rate under heat stress are due to a seasonal expansion of plasma volume [55,56], the active adolescents in the current study did not experience such a change (Table 2). As such their improved cardiovascular stability was likely mediated by the lower thermal strain experienced during the heat response test post-summer (i.e. lower exercising T_gi_ and T_sk_) and the earlier onset of sweating (~0.2°C). Our findings therefore indicate that thermal and cardiovascular adaptations are induced in adolescents completing ~7 h·wk^−1^ of outdoor physical activity in ~21.2°C WBGT.

In contrast, the summer months had a negligible effect on WBSL (~0.03 L), LSR at the back (~0.06 mg·cm^−2^·min^−1^) and forearm (~0.04 mg·cm^−2^·min^−1^), and thermosensitvity (~0.01 mg·cm^−2^·min^−1^·min^−1^), despite an earlier onset of sweating (~0.15°C), whilst a small reduction in sweat [Na^+^] was observed, primarily at the forearm (~8 mmol·L^−1^). The decrease in sweat [Na^+^] corresponds to an ~12% reduction, which is greater than what was observed in recreationally active adults in the same location (~9%) [54]. However, the 12% reduction is smaller than the average change documented in our systematic review (−23 ± 28%) [4] and is unlikely to meaningfully influence plasma osmolality. Previous investigations in adults have shown that seasonal heat acclimatization can enhance WBSR by 0.1–0.2 L·h^−1^ [56,57], but data regarding the adaptations of children and adolescents is sparse. Following a heat acclimation protocol, one study found that 9 to 12-year-old boys experienced a 0.11 L·h^−1^ increase in WBSR [9]. However, given that the thermal strain associated with heat acclimation is more consistent, controlled, and concentrated over a specific time frame (i.e. 38°C, 50% RH for 70 min and 6 days) than that of the summer months, it is not surprising that the change in WBSR (~0.05 L·h^−1^) in the current study is almost half that documented from an acclimation protocol. Moreover, in heat acclimatization studies conducted with adult populations exposed to a similar mean daytime WBGT as that of the current study (~21.2°C), including one in the same location, comparable changes in WBSR were reported (i.e. 0.03–0.06 L·h^−1^) [8,50,54]. As such, it does not appear that adolescents are less likely to adapt to seasonal changes in temperature as previously suggested [14], this notion is reaffirmed by the heat responses tests being uncompensable, suggesting that if enhancements in WBSR had occurred, they would have been able to be detected. Instead, the adaptive stimulus associated with exercising throughout a temperate summer (i.e. exercise frequency, intensity, duration and time of day) was likely not sufficiently stressful or equivalent to that of heat acclimation, to influence WBSR.

## Conclusion

To our knowledge, this is the first study to assess the magnitude of heat adaptations from the summer months in active adolescents. Our findings indicated that heat adaptations were induced in adolescents completing ~7 h·wk^−1^ of outdoor physical activity during a summer with a daytime WBGT of 21.2 ± 4.6°C. A reduction in resting T_gi_ and a lower T_gi_ at the onset of sweating, along with a lower exercising T_sk_, were associated with a smaller rise in T_gi_ during exercise in the heat. In contrast, changes in other sudomotor measures (i.e. WBSL, LSR and sweat [Na^+^]) were negligible, as were the changes in hematological (i.e. hemoglobin mass, plasma volume and blood volume) and perceptual (i.e. RPE, thermal sensation and thermal comfort) variables. Despite previous suggestions that pediatric populations adapt slower to the heat, and to a smaller magnitude than adults, heat acclimatization appears to have been induced similarly following a temperate summer in a group of active adolescents. However, future research is needed to directly compare the adaptation kinetics between pediatric and adult populations, while considering the influencing factors of seasonal heat acclimatization.

## List of abbreviatons

[Na^+^]: Sodium concentration
CO: Carbon Monoxide
CrI: Credible intervals
DXA: Dual-energy X-ray absorptiometry
HbCO: Carboxyhemoglobin
HGAM: Hierarchical generalized additive models
HRT: Heat response test
LSR: Local sweat rate
Pd: Probability of direction
RH: Relative humidity
RPE: Rating of perceived exertion
Tgi: Gastro-intestinal temperature
Tsk: Skin temperature
USG: Urine specific gravity
$\dot{V}$O2peak
Peak oxygen uptake
WBGT: Wet-bulb globe temperature
WBSL: Whole-body sweat loss
WBSR: Whole-body sweat rate

## Data Availability

The data and associated code used for analyses are available from the corresponding author upon reasonable request.
